# Cutaneous lesions in psoriatic arthritis are enriched in chemokine transcriptomic pathways

**DOI:** 10.1186/s13075-023-03034-6

**Published:** 2023-05-02

**Authors:** Hanna Johnsson, John Cole, Stefan Siebert, Iain B. McInnes, Gerard Graham

**Affiliations:** School of Infection and Immunity, University of Glasgow, 120 University Place, Glasgow, G12 8TA UK

**Keywords:** Psoriatic arthritis, Skin, Transcriptomics, Chemokines, Atypical chemokine receptor 2

## Abstract

**Objectives:**

Skin from people with psoriasis has been extensively studied and is assumed to be identical to skin from those with psoriatic arthritis (PsA). Chemokines and the CC chemokine scavenger receptor ACKR2 are upregulated in uninvolved psoriasis. ACKR2 has been proposed as a regulator of cutaneous inflammation in psoriasis. The aim of this study was to compare the transcriptome of PsA skin to healthy control (HC) skin and evaluate ACKR2 expression in PsA skin.

**Methods:**

Full-thickness skin biopsies from HC, lesional and uninvolved skin from participants with PsA were sequenced on NovaSeq 6000. Findings were validated using qPCR and RNAscope.

**Results:**

Nine HC and nine paired PsA skin samples were sequenced. PsA uninvolved skin was transcriptionally similar to HC skin, and lesional PsA skin was enriched in epidermal and inflammatory genes. Lesional PsA skin was enriched in chemokine-mediated signalling pathways, but uninvolved skin was not. ACKR2 was upregulated in lesional PsA skin but had unchanged expression in uninvolved compared with HC skin. The expression of ACKR2 was confirmed by qPCR, and RNAscope demonstrated strong expression of ACKR2 in the suprabasal layer of the epidermis in PsA lesions.

**Conclusion:**

Chemokines and their receptors are upregulated in lesional PsA skin but relatively unchanged in uninvolved PsA skin. In contrast to previous psoriasis studies, ACKR2 was not upregulated in uninvolved PsA skin. Further understanding of the chemokine system in PsA may help to explain why inflammation spreads from the skin to the joints in some people with psoriasis.

**Supplementary Information:**

The online version contains supplementary material available at 10.1186/s13075-023-03034-6.

## Background

Psoriasis is a common inflammatory skin condition and approximately 20% of people with psoriasis develop psoriatic arthritis (PsA) [1]. In most cases, the skin disease precedes the onset of arthritis. Risk factors reported to be associated with PsA include family history, psoriatic nail disease, obesity and trauma [2], but the mechanisms that lead to the development of PsA are not currently clear.

The transcriptomic profiles of lesional and uninvolved skin in people with psoriasis have been extensively studied using microarray, bulk tissue RNA sequencing (RNAseq) and recently single-cell RNAseq. These studies have identified dysregulation of IL-17 pathway genes, chemokines and other inflammatory and epidermal genes in both psoriasis lesions and uninvolved skin in people with psoriasis [3–10]. Some psoriasis skin transcriptomic studies included participants with PsA but did not differentiate between psoriasis and PsA in the analysis. However, a proteomic study did and identified differences between psoriasis and PsA skin [11].

Chemokines are *chemo*tactic cyto*kines* defined by conserved cysteine residues in their N-terminal end which delineates the four families of CC, CXC, C3XC and XC chemokines [12]. They bind to their canonical receptors, inducing migration of the cell expressing the receptor. They also bind to the atypical chemokine receptors (ACKR) which do not induce cellular migration. Chemokines can be split into homeostatic and inflammatory based on their functions. Typically, the inflammatory chemokine receptors have multiple ligands. The inflammatory CC chemokine scavenging receptor ACKR2 has been proposed as a regulator of cutaneous inflammation in psoriasis with a two-fold increased expression in psoriasis skin lesions and > tenfold upregulation in psoriasis uninvolved skin, with reduced expression following mild trauma [13].

In the present study, we performed bulk tissue RNAseq on lesional and uninvolved skin samples from people with PsA and compared this with results from people without psoriasis or PsA (healthy controls; HC). To the best of our knowledge, no published study to date has specifically investigated the transcriptome of skin in PsA. Here we show that uninvolved skin in PsA is transcriptionally very similar to HC skin with only 15 differentially expressed genes (DEGs). Transcriptomic changes in PsA skin lesions are similar to those previously described in psoriasis lesions and skin lesions are enriched in chemokine pathways. There was upregulation of chemokines associated with both the innate and adaptive immune system. ACKR2 was upregulated in lesional PsA skin but in contrast to previous psoriasis studies, its expression was unchanged in uninvolved skin compared with HC skin.

## Methods

### Participants and samples

Participants with PsA with active skin disease amenable to biopsy and not on biologic treatments were recruited from Rheumatology clinics in Glasgow, UK. They all had a PsA diagnosis made by a consultant rheumatologist and had peripheral arthritis. Patients with disease limited to axial or enthesial structures were excluded; HC were recruited from staff and postgraduate students at the University of Glasgow.

Control and PsA uninvolved skin biopsies were taken from the buttock area, and lesional biopsies were taken from within a skin lesion, 1 cm from the edge. Uninvolved skin was defined as at least 8 cm from a skin lesion. Each full-thickness, 6 mm punch biopsy was cut in half and the halves placed in either RNAlater (Ambion) for RNA extraction or 10% neutral buffered formalin (SIGMA) for tissue fixation, processing and embedding.

Written informed consent was obtained from all participants in accordance with the declaration of Helsinki, and the project was reviewed and approved by the West of Scotland Research Ethics Committee (reference 16/WS/0059).

### RNA extraction and DNAse treatment

Biopsies in RNAlater were cut into smaller pieces before they were disrupted and homogenised in RLT buffer (Qiagen, Manchester, UK) with added β-mercaptoethanol in a TissueLyser (Qiagen) at 50 Hz with steel beads. RNA was extracted from the lysate as per the RNeasy Mini RNA extraction kit (Qiagen) protocol. The extracted RNA was treated with DNase MAX (Qiagen) to remove genomic contamination.

### Library preparation

Polyadenylated RNA was selected using the NEBNext Poly(A) mRNA Magnetic Isolation Module kit (New England Biolabs, Hitchin, UK) to preferentially sequence mRNA. The NEBNext Ultra II Directional RNA Library Prep Kit with sample purification beads (New England Biolabs) was used for the subsequent library preparation and each sample was labelled using i5 and i7 primers from the NEBNext Multiplex Oligos for Illumina (Dual Index Primers Set 1) (New England Biolabs).

The samples were pooled, and quality controlled by Qubit and Bioanalyzer. Sequencing was performed at Edinburgh Genomics (https://genomics.ed.ac.uk/) on the NovaSeq 6000 S1 (Illumina) using paired-end sequencing of 50 base pairs with a total of 750 M + 750 M reads.

### RNAseq analysis

To process raw RNAseq datasets we used the following pipeline: Firstly, the FastQ files were QC’d using FastQC v0.11.7 [14], and then they were aligned to the reference genome using STAR v2.6 [15] with –quantMode GeneCounts, –outFilterMultimapNmax 1 and –outFilterMatchNmin 35. We used a Star index with a –sjdbOverhang of the maximum read length − 1. Next, read count files were merged and genes with mean of < 1 read per sample were excluded. Finally, the expression and differential expression values were generated using DESeq2 v1.24 [16]. For differential comparisons, we used an A versus B model with no additional covariates. All other parameters were left to default. Sequences were aligned to the genome and transcriptome GRCh38 (release 91).

The data was explored and visualised using Searchlight2 [17]. Specifying 3 differential expression workflows (PsA L vs HC, PsA U vs HC and PsA L vs PsA U) and one multiple differential expression workflow (PsA L vs HC + PsA U vs HC). The significance threshold for genes was set to p_adj_ = 0.05 and log2fold = 1. Overrepresentation analysis was included using the default human GO biological processes database [18], and a threshold for significant enrichment of p_adj_ = 0.05. All other parameters were left to default.

### Quantitative PCR

The High Capacity RNA-to-cDNA kit (Applied Biosystems) was used as per protocol, with 500 ng input RNA to convert RNA to cDNA for qPCR analysis. Sample cDNA and standard samples were mixed with SYBR Green Mix (Quanta), nuclease-free water and primers (ACKR2 F: AGGAAGGATGCAGTGGTGTC; R: CGGAGCAAGACCATGAGAAG; TATA-binding protein (TBP) F: AGGATAAGAGAGCCACGAACC; R: GCTGGAAAACCCAACTTCTG). They were run in triplicates on the QuantStudio Real-Time qPCR machine (Step 1: 95 °C 20 s; Step 2: 40 × (95 °C 1 s, 60 °C 20 s); melt curve: 95 °C 15 s, 60 °C 1 min, 95 °C 15 s). Relative gene expression was assessed using the standard curve as a reference, and all results were normalised to the expression of the housekeeping gene TBP.

### RNA in situ hybridisation (RNAscope)

Formalin-fixed, paraffin-embedded biopsies were cut at 5 microns. The RNAscope 2.5 HD Assay Red (ACD) protocol was followed using Probe-Hs-ACKR2. Slides were imaged on an EVOS (Thermofisher) microscope.

### Statistical analysis

Analysis of RNAseq data has been described above. Statistical analysis comparing participant characteristics and gene expression of specific genes was carried out in GraphPad Prism version 9.0.0 for Windows (GraphPad Software, San Diego, CA, USA). Groups were compared using *t*-test, paired *t*-test or ANOVA with multiple comparisons. Non-parametric data were transformed prior to analysis. Correlation was assessed using Spearman’s correlation coefficient.

## Results

### Participant characteristics

Nine HC and nine participants with PsA were recruited. The participant characteristics are summarised in Additional file 1. The median (25th; 75th) tender joint count was 2 (0.5; 7), and the median (25th; 75th) swollen joint count was 1 (0; 1.5). Their median (25th; 75th) PASI score was 5.3 (5.2; 10.8), indicating mild to moderate skin disease activity. Although the participants in the PsA cohort were older than HCs, this was not significant (*p* 0.1257). The time since diagnosis ranged from < 1 year to 14 years.

### PsA lesional skin is transcriptionally distinct from HC and uninvolved skin

**Fig. 1:**
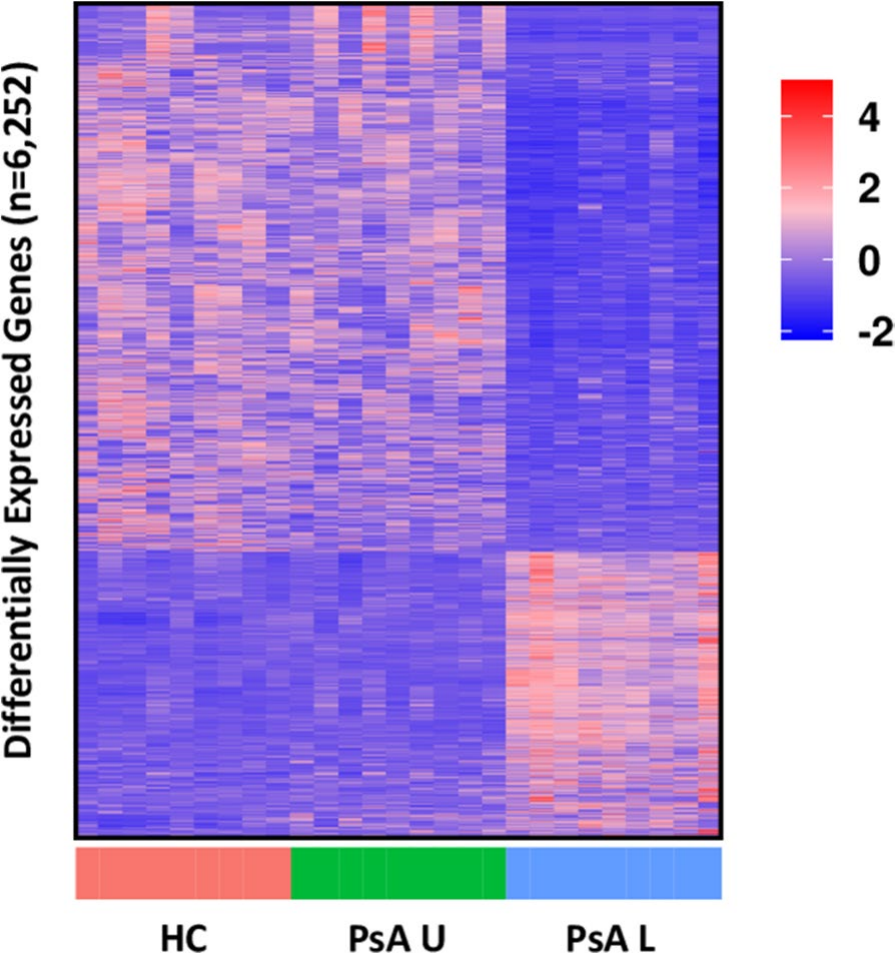
PsA uninvolved and HC skin are transcriptionally similar, but PsA skin lesions are transcriptionally distinct. Expression heatmap of genes that were significantly different (p_adj_ < 0.05, absolute log_2_fold > 1) between healthy control skin (HC) and PsA uninvolved skin (PsA U) or PsA lesional skin (PsA L). Genes are given on the y-axis and samples on the *x*-axis. The *y*-axis has been hierarchically clustered. Expression values are given as per gene *Z*-scores, with high = red and low = blue

There were 15 significant DEGs when PsA uninvolved skin was compared to HC skin, and 6245 significant DEGs when PsA skin lesions were compared to HC skin. The expression of all significant DEGs in all samples was summarised in a heatmap (Fig. 1). PsA lesional samples appeared distinct from HC and samples from uninvolved skin in PsA, with similar gene expression in HC and uninvolved samples. Of the 15 significant DEGs in PsA uninvolved skin compared to HC skin, 10 were also significant DEGs in PsA lesional skin compared to HC skin (Additional file 2). These genes were not associated with enrichment of any specific biological pathways.

### PsA lesions display changes in keratinisation

Comparing PsA skin lesions to HC skin indicated 4094 downregulated and 2151 upregulated DEGs in PsA lesions. Hypergeometric gene set enrichment analysis of all DEGs using the gene ontology biological process (GO_BP) database identified ‘Keratinization’ as the most significantly enriched pathway (log2fold enrichment 1.8587, p_adj_ 4.7259E − 13). The enrichment was due to both upregulated (n = 23) and downregulated genes (n = 15) (Additional file 3). The DEGs in this pathway included 16 late cornified envelopes, 10 small proline-rich proteins and three keratin genes.

### Downregulated DEGs in PsA skin lesions are enriched in lipid metabolism genes

**Fig. 2:**
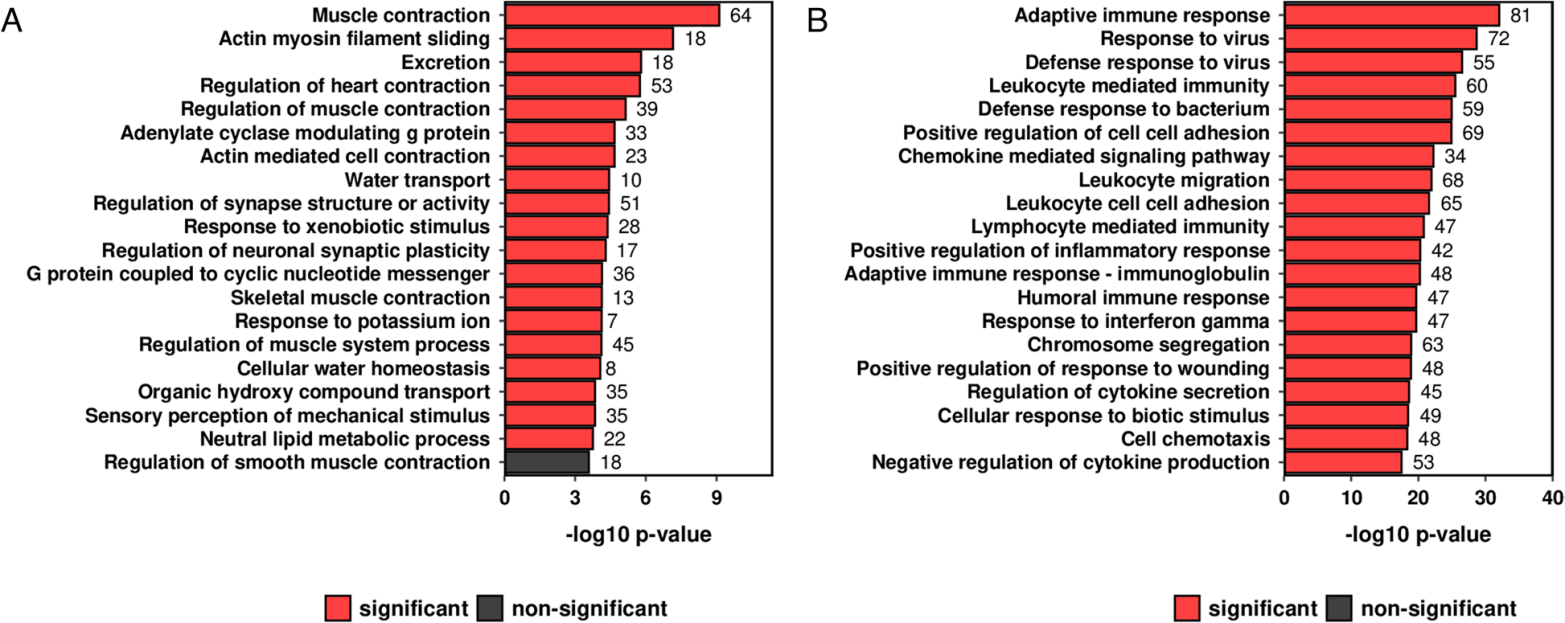
The 20 most enriched gene sets for PsA lesions vs HC. The 20 most enriched gene sets (Gene-Ontology, overrepresentation analysis, hypergeometric test) when PsA skin lesions were compared with healthy control skin. For **a** significantly (p_adj_ < 0.05, absolute log_2_fold > 1) downregulated genes and **b** significantly upregulated genes. The enrichment adjusted *p*-value (-log_10_) is given on the *x*-axis and gene-set name on the *y-axis*. Gene sets that were significantly enriched (p_adj_ < 0.05) are shown in red. The data labels denote the number of differentially expressed genes within each gene set

The 25 most downregulated genes in PsA lesional skin compared with HC skin are presented in Table 1. Nine of these lacked a descriptive name, and four genes were non-protein coding (LINC02169, IL12A-AS1, LINC02527, HSD3BP2). Among protein-coding genes, several lipid metabolism genes (MOGAT1, AADACL3, THRSP, PM20D1, FADS2) and sebaceous gland genes (AWAT1, AWAT2 and DGAT2L6) were identified; in addition, four of the genes without a descriptive name are expressed in sebaceous glands [19, 20]. Consistent with these observations, the ‘Neutral lipid metabolic process’ biological pathway was enriched in lesional skin (log2fold enrichment 1.1533, p_adj_ 0.0373). It was one of 19 enriched pathways among downregulated genes in PsA skin lesions (Fig. 2a). The most enriched pathway is related to muscle contraction. There was also an overrepresentation of genes relating to G-protein coupled receptors, synapse, excretion, and water homeostasis among downregulated DEGs.

**Table 1:** The 25 most downregulated genes in PsA lesional skin compared with healthy control skin

Symbol	Gene name	PsA lesional skin vs HC skin	
		**Log2fold**	**Padj**
**LINC02169**	Long intergenic non-protein coding RNA 2169	− 8.5	3.87E − 11
**DGAT2L6**	Diacylglycerol O-acyltransferase 2 like 6	− 7.75	2.75E − 12
**MOGAT1**	Monoacylglycerol O-acyltransferase 1	− 7.72	2.41E − 08
**IL12A-AS1**	IL12A antisense RNA 1	− 7.67	1.21E − 06
**AADACL3**	Arylacetamide deacetylase like 3	− 7.62	4.01E − 13
**AC112243.1**	NA	− 7.51	6.28E − 10
**AGR3**	Anterior gradient 3, protein disulphide isomerase family member	− 7.22	1.33E − 16
**AL513321.2**	NA	− 7.21	2.4E − 18
**GAL**	Galanin and GMAP prepropeptide	− 7.12	1.93E − 12
**AC138647.1**	NA	− 7	8.25E − 15
**AWAT2**	Acyl-CoA wax alcohol acyltransferase 2	− 6.98	1.37E − 11
**C1ORF158**	Chromosome 1 open reading frame 158	− 6.93	8.08E − 05
**LINC02527**	Long intergenic non-protein coding RNA 2527	− 6.8	4.07E − 15
**AC022784.6**	NA	− 6.65	4.97E − 17
**THRSP**	Thyroid hormone responsive	− 6.64	2.37E − 13
**AC109462.2**	NA (cluster0007)^a^	− 6.57	7.31E − 06
**PM20D1**	Peptidase M20 domain containing 1	− 6.55	6.44E − 12
**HSD3BP2**	Hydroxy-delta-5-steroid dehydrogenase, 3 beta, pseudogene 2	− 6.5	1.18E − 05
**AWAT1**	Acyl-CoA wax alcohol acyltransferase 1	− 6.45	2.05E − 08
**AL158817.1**	NA (cluster0007)^a^	− 6.38	7.5E − 05
**TRIM55**	Tripartite motif-containing 55	− 6.34	6.65E − 09
**AC110009.1**	NA (cluster0007)^a^	− 6.2	1.07E − 05
**AC091163.1**	NA (cluster0007)^a^	− 6.05	4.65E − 06
**AP001330.1**	NA	− 6.04	2.64E − 15
**ZNF725P**	Zinc finger protein 725, pseudogene	− 5.95	3.65E − 05

*HC* healthy control, *NA* not available, *padj* adjusted *p*-value, *PsA* psoriatic arthritis

^a^Cluster0007 refers to a sebaceous gland gene cluster identified by Shih et al. [20]

### Epidermal and inflammatory genes are upregulated in PsA skin lesions

The 25 most upregulated genes in PsA skin lesions compared with HC skin are presented in Table 2. They can broadly be grouped as cornified envelope genes (small proline-rich proteins SPRR2A, SPRR2C, SPRR2F and late cornified envelope gene LCE3A), antimicrobial peptides (defensins DEFB4A, DEFB4B, S100 calcium-binding proteins S100A7A, S100A9, S100A12 and peptidase inhibitor 3 PI3), interleukins (IL-36A, IL-19) and chemokines (CXCL1, CXCL6, CXCL8). These genes have previously been implicated in psoriasis pathogenesis [3, 21, 22].

**Table 2:** The 25 most upregulated genes in PsA lesional skin compared with healthy control skin

Symbol	Gene name	PsA lesional skin vs HC skin	
		**Log2fold**	**Padj**
**SPRR2C**	Small proline-rich protein 2C (pseudogene)	11.36	2.15E − 37
**IL36A**	Interleukin-36 alpha	10.63	2.45E − 38
**DEFB4A**	Defensin beta 4A	10.38	2.36E − 61
**DEFB4B**	Defensin beta 4B	9.65	3.39E − 11
**SPRR2F**	Small proline-rich protein 2F	9.18	2.99E − 69
**S100A7A**	S100 calcium-binding protein A7A	9.06	1.34E − 50
**CLEC3A**	C-type lectin domain family 3 member A	8.93	1.03E − 10
**PI3**	Peptidase inhibitor 3	8.78	8.4E − 115
**HSPD1P3**	NA	8.65	1.75E − 14
**CXCL8**	C-X-C motif chemokine ligand 8	8.56	1.95E − 37
**IGHV3-30**	Immunoglobulin heavy variable 3–30	8.4	8.98E − 06
**TCN1**	Transcobalamin 1	8.38	7.3E − 100
**LCE3A**	Late cornified envelope 3A	8.28	1.39E − 60
**IL19**	Interleukin-19	8.28	5.36E − 14
**SPRR2A**	Small proline-rich protein 2A	8.05	1.19E − 35
**KRT24**	Keratin 24	7.98	3.94E − 11
**CXCL6**	C-X-C motif chemokine ligand 6	7.89	3.07E − 09
**S100A12**	S100 calcium-binding protein A12	7.83	8.7E − 37
**ADGRF1**	Adhesion G protein-coupled receptor F1	7.79	1.97E − 48
**SERPINB4**	Serpin family B member 4	7.73	1.01E − 28
**TNIP3**	TNFAIP3 interacting protein 3	7.57	8.59E − 52
**S100A9**	S100 calcium-binding protein A9	7.56	4.27E − 31
**SPRR3**	Small proline-rich protein 3	7.52	4.52E − 09
**CXCL1**	C-X-C motif chemokine ligand 1	7.47	1.39E − 22

*HC* healthy control, *NA* not available, *padj* adjusted *p*-value, *PsA* psoriatic arthritis

### PsA skin lesions are enriched in chemokine pathways

**Fig. 3:**
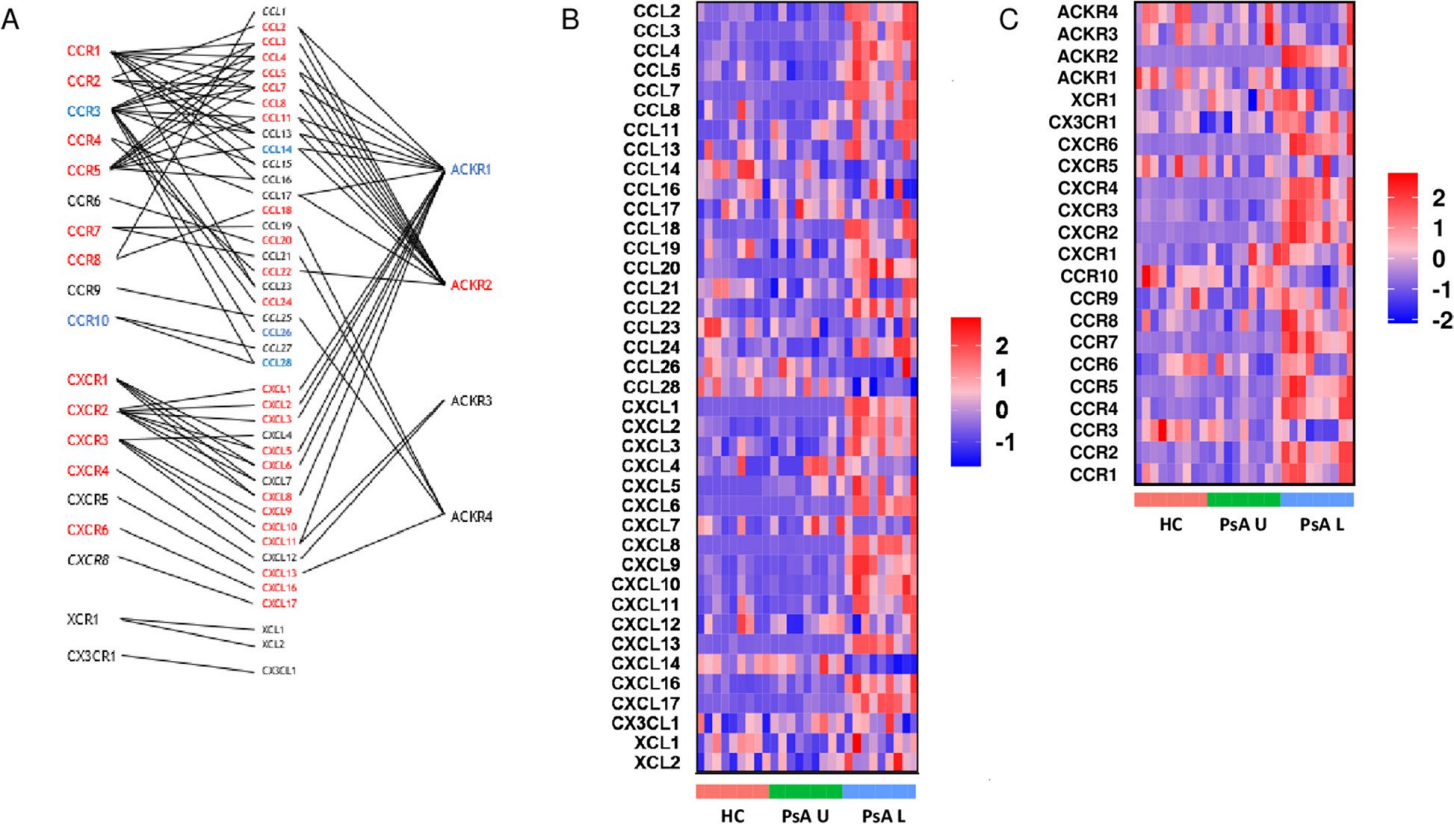
Inflammatory and homeostatic chemokine ligands and receptors are upregulated in PsA lesional skin. **a** Chemokine ligand and receptor interactions are indicated with lines. Red font indicates receptors and ligands which are transcriptionally upregulated in PsA skin lesions compared to healthy controls, and blue font indicates receptors and ligands which are transcriptionally downregulated in PsA skin lesions. Receptors and ligands which were not included in the transcriptional analysis are indicated by cursive writing. Expression heatmap of **b** chemokines and **c** chemokine receptors. Genes are given on the *y*-axis and samples on the *x*-axis. Expression values are given as per gene *Z*-scores, with high = red and low = blue

Significantly upregulated DEGs in PsA skin lesions were enriched for 662 GO_BP pathways. Half of the 20 most significantly enriched pathways (Fig. 2b) are related to chemokine signalling and cell migration. Chemokines and their receptors were therefore investigated further. Chemokine ligand and receptor interactions are shown in Fig. 3a, and the colour represents changes in expression in PsA skin lesions compared with HC skin (red = upregulated, blue = downregulated). The expression of chemokine and chemokine receptors are summarised as heatmaps in Fig. 3b and3c, respectively, and presented in Additional files 4 and 5.

There was upregulation of both inflammatory and homeostatic chemokines and receptors in lesional skin in PsA. The inflammatory receptors CCR1, CCR2, and CCR5, which are found on monocytes, monocyte-derived cells and some T cells [23–25], were all upregulated, as were most of their ligands. The neutrophil chemokine receptors CXCR1 and CXCR2 [12] were both upregulated. There was strong upregulation of most neutrophil chemoattracting chemokines, including the four most upregulated chemokine ligands in PsA lesions (CXCL1, CXCL5, CXCL6, CXCL8), suggesting an innate component to the inflammatory response in skin lesions. In contrast, the eosinophil receptor CCR3 and its ligands CCL26 and CCL28 were downregulated in lesional skin in PsA.

There were also differentially expressed chemokines and receptors linking the innate and adaptive immune response in skin lesions. The homeostatic receptor CCR7, which directs cells to lymphatics [26, 27], was upregulated, as was its ligand CCL19, but not CCL21. This is consistent with previous studies which identified increased CCL19 and CCR7 expression in dermal aggregates in psoriasis skin lesions [28, 29]. CXCL13, which directs CXCR5 expressing B cells and follicular helper T cells to the B cell zone in secondary lymphoid organs [30, 31], was significantly increased in skin lesions. The expression of CXCR5 was however unchanged.

The most upregulated chemokine receptor, by log2fold change, in PsA skin lesions was CXCR6 (log2fold 3.8, p_adj_ 1.32E − 26), and its expression correlated with PASI score (Additional file 6; Spearman *r* 0.7748, *p* 0.0492). CXCR6 is a receptor found on T cells, including cytotoxic CD8^+^ T, NK and plasma cells [12, 32, 33]. The transcription of its ligand CXCL16 was also increased in PsA skin lesions. CXCR3, another chemokine receptor of the adaptive immune system and expressed by Th1 T cells and a subset of memory B cells [12, 34, 35], was upregulated in skin lesions, as was the expression of its ligands CXCR9 and CXCR10, and to a lesser degree CXCR11. CCR6, which is expressed by IL-17-producing T cells [36], was unchanged but its ligand CCL20 was significantly upregulated in PsA skin lesions.

CCR4, which is expressed on Th2 T cells, regulatory T cells and cutaneous lymphocyte-associated antigen (CLA) positive, skin-homing T cells, was upregulated in skin lesions along with its ligand CCL22. CCR4^+^ CLA^+^ cells have previously been found in psoriasis skin lesions [34, 37]. CCR8 was recently identified as a marker of tissue-resident memory T cells in human skin [38]. The receptor was upregulated in PsA skin lesions, and its ligand CCL18 was the most upregulated CC chemokine by log2fold (log2 fold 5.03, p_adj_ 1.23E-08). CCR10, which is a further skin-homing receptor for memory-like T cells [12], was significantly downregulated in PsA lesions. There was concomitant downregulation of its ligand CCL28. Its second ligand, CCL27, was not included in the transcriptomic analysis.

### ACKR2 is upregulated in PsA skin lesions but not in PsA uninvolved skin

The atypical chemokine receptor ACKR2, which is expressed by stromal cells and scavenges inflammatory CC chemokines, was the most significantly upregulated chemokine receptor in PsA skin lesions (log2fold 3.38, p_adj_ 9.51E − 41, Fig. 3a and c). ACKR2 expression was not significantly changed in PsA uninvolved skin compared to HC skin (log2fold − 0.2, p_adj_ 0.7324). The increased ACKR2 expression in PsA skin lesions with unchanged expression in PsA uninvolved skin compared to HC skin was unexpected, as this contrasts with previous work in psoriasis skin, which showed strong upregulation in uninvolved skin in psoriasis [13].

**Fig. 4:**
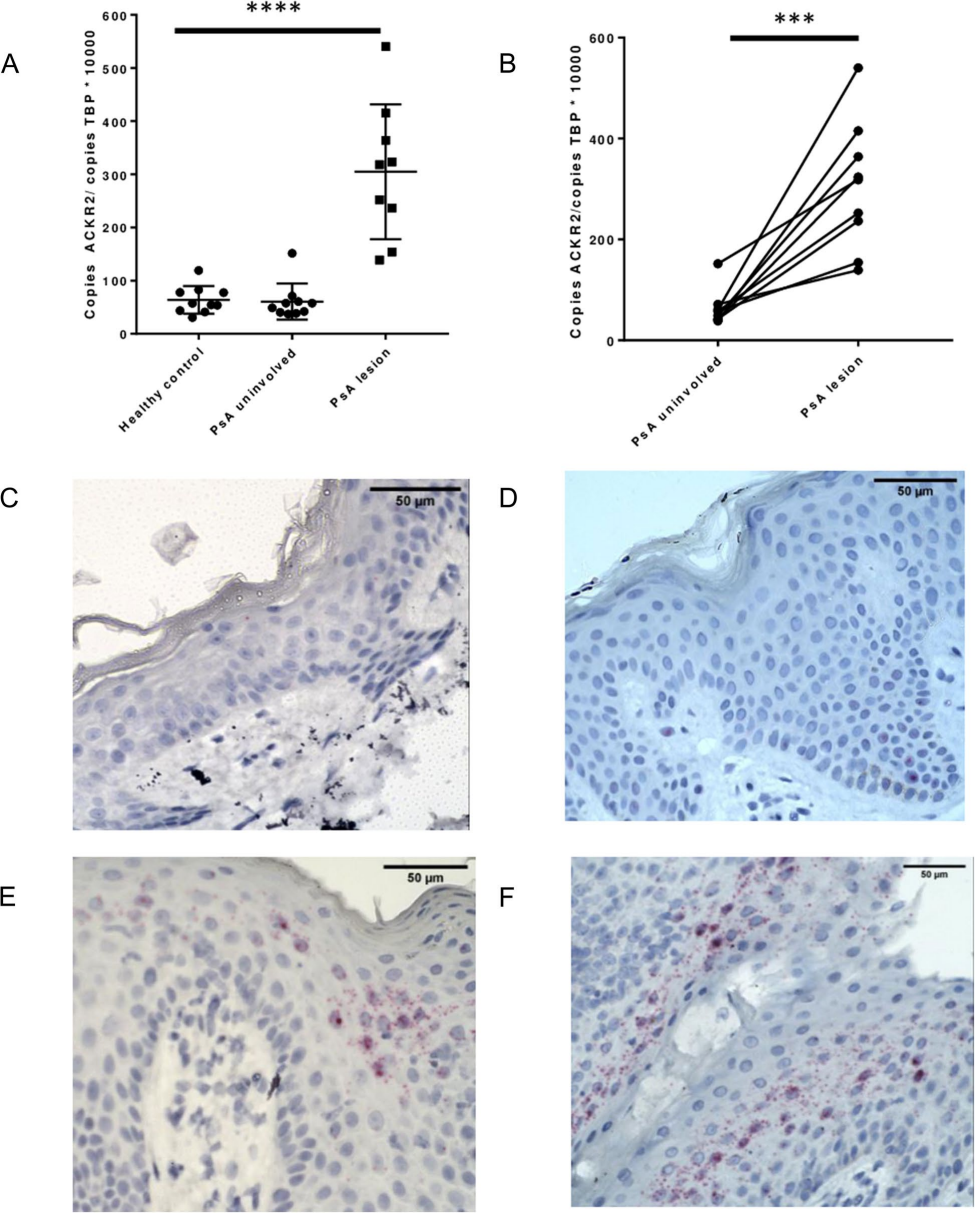
ACKR2 is upregulated in PsA skin lesions with overexpression in the suprabasal epidermis. Relative ACKR2 mRNA expression normalised to TATA-binding protein in full-thickness skin biopsies from **a** patients with PsA and healthy controls and **b** in paired samples of uninvolved and lesional skin from participants with PsA. Skin samples were probed for ACKR2 by RNAScope and detected with Red detection agent. **c** HC, **d** PsA uninvolved and **e**–**f** PsA lesional skin. ACKR2-positive cells stain red. Scale bars 50 μm. *** *p* 0.0006, **** *p* < 0.0001

The PsA skin RNAseq findings were confirmed by qPCR analysis (Fig. 4a), and ACKR2 expression was higher in the skin lesion compared to the paired uninvolved skin in all participants (Fig. 4b). Consistent with this, weak staining was seen in HC and uninvolved samples using RNA in-situ hybridisation (Fig. 4c, d) with strong staining in the suprabasal epidermis of PsA lesional skin (Fig. 4e, f).

## Discussion

This paper has presented an analysis of bulk tissue transcriptional changes in paired uninvolved and lesional skin from participants with PsA for the first time. The results are largely concordant with previous transcriptomic studies in psoriasis skin, with enrichment of pathways relating to keratinization, innate and adaptive immune responses, chemotaxis, and leukocyte migration. Many of the most upregulated genes in PsA skin lesions are upregulated in both lesional and uninvolved skin in psoriasis [3, 21, 22]. In contrast, only a small number of significant DEGs were identified in uninvolved PsA skin.

Sebaceous gland and lipid metabolism genes were among downregulated genes in PsA lesional skin compared to HC skin. These genes have previously also been found to be downregulated in psoriasis lesions [39, 40]. Moreover, the lipid metabolism genes THRSP and GAL, which are expressed by keratinocytes and in eccrine sweat glands [41], have also been reported to be downregulated in uninvolved psoriasis skin [40]. Here both were downregulated in PsA lesions but not in PsA uninvolved skin.

The most enriched pathway among downregulated genes related to muscle contraction; these pathways have been identified by others as enriched among downregulated DEGs in psoriasis skin lesions [4, 9]. They are dermally expressed genes and likely to be downregulated as the dermis makes up a smaller proportion of lesional skin biopsies than in normal skin due to the expanded epidermis.

Among upregulated genes in PsA lesions compared to HC skin, there was an enrichment of chemokine genes. The most upregulated chemokines were the neutrophil-attracting chemokines. This result is consistent with the known abundance of neutrophils in psoriatic skin lesions [42]. Moreover, a neutrophil activation signature has been identified in blood in patients with psoriasis [43], and higher expression of CXCR1 and neutrophil elastase are present in synovial tissue in PsA compared to rheumatoid arthritis [44]. Furthermore, CXCL8 is induced to a greater extent in synovial fibroblasts from patients with PsA than in rheumatoid arthritis [44].

The most upregulated chemokine receptor in PsA skin lesions by log2fold was CXCR6, with its expression positively correlated with the PASI score. CXCR6^+^ CD8^+^ T cells are found in the dermis and epidermis in psoriasis but not atopic dermatitis skin lesions [32]. The CXCR6 receptor is also found on the majority of synovial Th17^+^ CD8^+^ cells, the majority of which also express CCR6 [33]. Micro-array analysis of synovial membrane identified upregulation of both CCR6 and its ligand CCL20 in PsA compared to HC [45]. In this study, the expression of CCR6 in PsA skin lesions was unchanged but its ligand CCL20 was strongly upregulated.

The scavenging receptor ACKR2 has previously been identified as a potential regulator of cutaneous inflammation in psoriasis with a two-fold increased expression in psoriasis skin lesions and > tenfold upregulation in psoriasis uninvolved skin [13]. Previous work demonstrated that the expression of ACKR2 in uninvolved skin in psoriasis could be reduced by mild trauma, representing a potential mechanism for the Koebner phenomenon observed in this condition [13]. Moreover, induction of ACKR2 expression by systemic IFNγ treatment in mice reduced the psoriatic severity score and recruitment of CD3^+^ T cells to imiquimod-treated skin [46]. In *Ackr2*^*−/−*^ mice there were increased numbers of CD3^+^ T cells in the psoriasiform epidermis in mice compared to wild-type mice following imiquimod treatment. It was therefore surprising that the expression of ACKR2 in PsA uninvolved skin was unchanged and strongly upregulated in PsA skin lesions. The distribution of ACKR2-expressing cells PsA skin was also different to previous observations in psoriasis; the current study identified sparse expression in HC skin with a similar pattern in PsA-uninvolved skin. This contrasts with extensive ACKR2 expression throughout the epidermis in uninvolved psoriasis skin in Singh et al.’s study [13]. In psoriasis lesions, there was also expression of ACKR2 throughout the epidermis, but in our PsA lesional samples, most ACKR2-expressing cells were found in the suprabasal epidermis. The current study identified ACKR2-expressing cells in PsA skin using RNAscope while Singh et al. used ACKR2 antibodies. ACKR2 protein has a long half-life, which may contribute to the differences observed. Moreover, it is not known from which anatomical site biopsies were taken in Singh et al.’s study. This may be relevant as gene expression in skin is different at different anatomical locations [47]. It is also not known what treatments the patients with psoriasis received. No participant in the current study was receiving biologic treatment or phototherapy. The findings do however suggest a potential differential role of ACKR2 in psoriasis and PsA skin, and it could be postulated that a dysregulation of ACKR2 in PsA skin may allow the spread of inflammation beyond the skin.

However, the study is limited by the small sample number and the lack of a direct comparison between the skin with people with psoriasis with and without arthritis. Moreover, 5 of 9 participants received systemic treatment, and most participants had mild psoriasis and the articular domain was in remission in some participants. These factors may all have influenced the results.

## Conclusions

This study has identified dysregulation of the expression of chemokine genes of both the innate and adaptive immune system in PsA skin lesions. Further studies are warranted to compare transcriptional differences between psoriasis and PsA skin and to evaluate how the expression of chemokines may contribute to the distribution of inflammatory disease in PsA.

## Supplementary Information

**Additional file 1.** Participant characteristics.

**Additional file 2.** Significantly differentially expressed genes in PsA uninvolved skin compared to HC skin.

**Additional file 3.** Differentially expressed genes in the GO Keratinization gene set. Boxplots of DEGs (p_adj_ <0.05, absolute log_2_fold >1) which form part of the GO Keratinization gene set when PsA skin lesions (PsA L) were compared to healthy control (HC) skin. Genes are given on the x-axis and expression values (per gene z-scores) on the y-axis.

**Additional file 4.** Expression of chemokine ligands in skin.

**Additional file 5. **Expression of chemokine receptors in skin.

**Additional file 6.** The expression of CXCR6 in skin lesions correlates with PASI score. The normalised read counts of CXCR6 are plotted on the y-axis and PASI scores on the x-axis. Spearman r 0.7748, p 0.04921.

## Data Availability

The dataset generated during the current study are available in the Gene Expression Omnibus (GEO) repository, under accession GSE205748. https://www.ncbi.nlm.nih.gov/geo/query/acc.cgi?acc=GSE205748

## Abbreviations

ACKR: Atypical chemokine receptor
CLA: Cutaneous lymphocyte-associated antigen
DEG: Differentially expressed gene
GO_BP: Gene ontology biological process
HC: Healthy control
p_adj_: Adjusted *p*-value
PsA: Psoriatic arthritis
RNAseq: RNA sequencing
TBP: TATA-binding protein
