# Supplementary material for: Cutaneous lesions in psoriatic arthritis are enriched in chemokine transcriptomic pathways
Source: Arthritis Res Ther. 2023 May 2;25:73. doi: 10.1186/s13075-023-03034-6 (PMC10152590; doi:10.1186/s13075-023-03034-6)
Supplement: Supplementary file 1 — Additional file 1. Participant characteristics. [file 13075_2023_3034_MOESM1_ESM.pdf]

	**PsA (n=9)**	**HC (n=9)**
**Mean age,** *years* (SD)	46.44 (16.27)	36.44 (8.95)
**Time since PsA diagnosis**<br>• 0-4 years<br>• 5-9 years<br>• >10 years	 4<br>1<br>4	
**Female gender**, *n* (%)	5 (55.6%)	6 (66.7%)
**Tender joint count** *median* (25th; 75th)	2 (0.5; 7)	
**Swollen joint count** *median* (25th; 75th)	1 (0; 1.5)	
**PASI** *median* (25th; 75th)[a]	5.3 (5.2; 10.8)	
**Body surface area (%)** *median* (25th; 75th)[b]	6.75 (3.75; 13.75)	
**CRP (mg/L),** *mean* (SD)	10.14 (9.63)	
**ESR (mm/hr)** *mean* (95% CI)	10.38 (8.12)	
**DMARD** treatment (n)<br>• No DMARD<br>• Methotrexate<br>• Apremilast	 4<br>3<br>2	

Additional file 1. Participant characteristics

CRP, C-reactive protein; DMARD, disease modifying antirheumatic drug; ESR erythrocyte sedimentation rate; PASI psoriasis area severity index.

[a]Data missing for 2 participants

[b]Data missing for 1 participant
