# Supplementary material for: Cutaneous lesions in psoriatic arthritis are enriched in chemokine transcriptomic pathways
Source: Arthritis Res Ther. 2023 May 2;25:73. doi: 10.1186/s13075-023-03034-6 (PMC10152590; doi:10.1186/s13075-023-03034-6)
Supplement: Supplementary file 2 — Additional file 2. Significantly differentially expressed genes in PsA uninvolved skin compared to HC skin. [file 13075_2023_3034_MOESM2_ESM.pdf]

Symbol	Gene name	PsA Uninvolved skin vs HC skin		PsA Lesional skin vs HC skin	
		Log2fold	p.adj	Log2fold	p.adj
**RPL35P5**	ribosomal protein L35 pseudogene 5	-1.73	0.0498	-1.87	0.0003
**SNORA73B**	small nucleolar RNA, H/ACA box 73B	-1.63	0.0063	0.7	0.0311
**SNHG25**	small nucleolar RNA host gene 25	-1.1	0.028	-0.64	0.0395
**PCP2**	Purkinje cell protein 2	-1.04	0.0498	-2.11	0
**SMKR1**	small lysine rich protein 1	-1.03	0.0443	-0.47	0.1222
**AC010300.1**	NA	1.09	0.028	-1.38	0
**Z82217.1**	NA	1.26	0.046	-0.25	0.6064
**MYO3B**	myosin IIIB	1.35	0.0063	-1.47	0
**AC116407.1**	NA	1.82	0.028	0.18	0.7849
**TF**	transferrin	2.18	0.028	0.65	0.3285
**KLHDC7A**	kelch domain containing 7A	2.93	0.0063	2.66	0
**EGFL6**	EGF like domain multiple 6	3.04	0.0078	1.81	0.0174
**SULT4A1**	sulfotransferase family 4A member 1	3.18	0.0078	0.6	0.5124
**SPP1**	secreted phosphoprotein 1, osteopontin	3.44	0.0145	2.32	0.0091
**C21ORF33**	chromosome 21 open reading frame 33	4.74	0.0249	4.3	0.0005

Additional file 2. Significantly differentially expressed genes in PsA uninvolved skin compared to HC skin.

HC, healthy control; NA, not available; padj, adjusted p-value; PsA psoriatic arthritis
