# Supplementary material for: Cutaneous lesions in psoriatic arthritis are enriched in chemokine transcriptomic pathways
Source: Arthritis Res Ther. 2023 May 2;25:73. doi: 10.1186/s13075-023-03034-6 (PMC10152590; doi:10.1186/s13075-023-03034-6)
Supplement: Supplementary file 3 — Additional file 3. Differentially expressed genes in the GO Keratinization gene set. Boxplots of DEGs (padj <0.05, absolute log2fold >1) which form part of the GO Keratinization gene set when PsA skin lesions (PsA L) were compared to healthy control (HC) skin. Genes are given on the x-axis and expression values (per gene z-scores) on the y-axis. [file 13075_2023_3034_MOESM3_ESM.zip › Additional file 3.pdf]

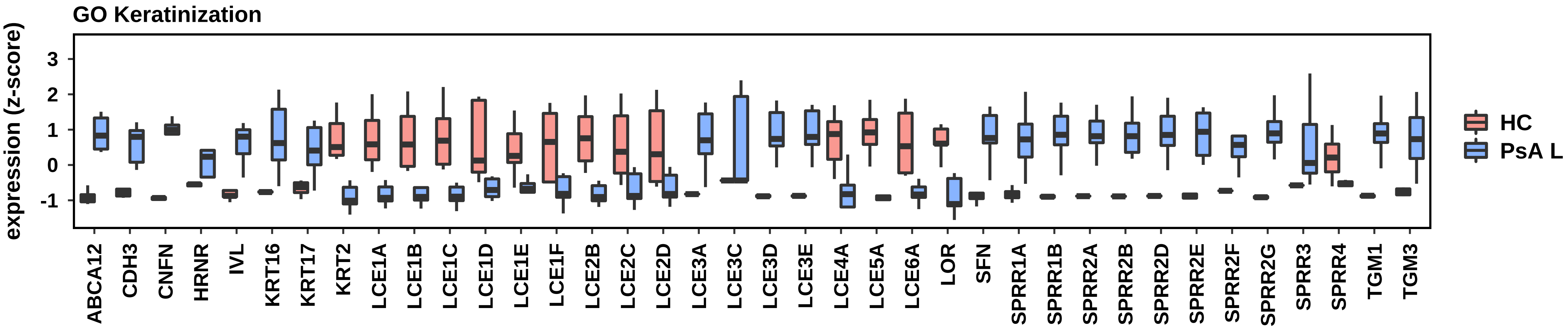

Additional file 3. Differentially expressed genes in the GO Keratinization gene set.
Boxplots of DEGs (p_{adj} <0.05, absolute log_2fold >1) which form part of the GO Keratinization gene set when PsA skin lesions (PsA L) were compared to healthy control (HC) skin. Genes are given on the x-axis and expression values (per gene z-scores) on the y-axis.
