# Supplementary material for: Cutaneous lesions in psoriatic arthritis are enriched in chemokine transcriptomic pathways
Source: Arthritis Res Ther. 2023 May 2;25:73. doi: 10.1186/s13075-023-03034-6 (PMC10152590; doi:10.1186/s13075-023-03034-6)
Supplement: Supplementary file 4 — Additional file 4. Expression of chemokine ligands in skin. [file 13075_2023_3034_MOESM4_ESM.pdf]

	Mean normalised read count			PsA U vs HC		PsA L vs HC	
**symbol**	**HC**	**PsA U**	**PsA L**	**log2fold**	**p.adj**	**log2fold**	**p.adj**
**CCL2**	229.70	178.42	706.92	-0.36	0.5648	1.62	3.11E-07
**CCL3**	0.23	0.20	6.14	-0.01	NA	4.43	2.5E-05
**CCL4**	7.36	4.76	53.76	-0.64	0.5620	2.87	1.51E-08
**CCL5**	89.21	76.14	242.08	-0.23	0.8001	1.44	0.00043
**CCL7**	0.50	0.00	16.19	-1.58	NA	4.9	7.61E-09
**CCL8**	9.89	5.98	28.92	-0.74	0.5386	1.53	0.013152
**CCL11**	0.20	0.78	3.75	1.43	NA	3.73	0.00214
**CCL13**	169.03	138.96	429.86	-0.28	0.8625	1.35	0.068634
**CCL14**	72.20	42.65	33.79	-0.76	0.1913	-1.1	0.000824
**CCL16**	5.34	5.28	4.93	-0.03	NA	-0.14	0.833048
**CCL17**	25.27	47.53	29.37	0.91	0.4033	0.22	0.776214
**CCL18**	36.00	52.57	1175.70	0.55	0.7858	5.03	1.23E-08
**CCL19**	377.88	261.45	474.59	-0.53	0.4219	0.33	0.428909
**CCL20**	13.28	11.71	119.00	-0.18	0.9191	3.16	3.22E-06
**CCL21**	1037.53	820.09	1028.30	-0.34	0.4528	-0.01	0.96874
**CCL22**	175.51	174.16	770.78	-0.01	0.9926	2.13	1.43E-09
**CCL23**	17.91	11.52	10.82	-0.65	NA	-0.74	0.151156
**CCL24**	16.38	10.00	45.01	-0.71	0.49680	1.45	0.00806
**CCL26**	13.05	11.49	62.55	-0.19	NA	-1.48	0.000546
**CCL28**	357.56	327.89	254.39	-0.12	0.6914	-0.49	0.001439
**CXCL1**	2.93	4.47	525.28	0.58	0.7467	7.47	1.39E-22
**CXCL2**	7.63	9.95	65.15	0.41	0.7314	3.12	2.47E-09
**CXCL3**	3.92	2.52	17.12	-0.64	NA	2.1	0.000519
**CXCL4**	2.53	5.73	2.58	1.18	NA	0.03	0.980566
**CXCL5**	0.11	2.31	23.93	3.34	NA	6.72	1.41E-07
**CXCL6**	0.10	0.44	54.09	0.95	0.7830	7.89	3.07E-09
**CXCL7**	5.23	13.77	7.09	1.4	NA	0.45	0.684414
**CXCL8**	4.08	6.63	1537.43	0.7	0.6386	8.56	1.95E-37
**CXCL9**	53.87	50.41	631.39	-0.1	0.9574	3.55	8.94E-09
**CXCL10**	22.38	17.26	255.34	-0.38	0.8080	3.51	1.67E-07
**CXCL11**	7.28	5.93	42.44	-0.31	0.8586	2.53	0.000432
**CXCL12**	5314.94	5629.02	5722.97	0.08	0.9142	0.11	0.787808
**CXCL13**	2.44	1.12	227.06	-1.08	0.6098	6.55	7.43E-13
**CXCL14**	43472.46	45057.94	26538.43	0.05	0.8826	-0.71	6.56E-07
**CXCL16**	879.28	952.36	1793.27	0.12	0.6910	1.03	2.52E-14
**CXCL17**	3.39	5.10	41.74	0.6	0.5452	3.63	1.14E-16
**CX3CL1**	530.11	603.12	560.91	0.19	0.5057	0.08	0.650601
**XCL1**	8.19	3.62	8.77	-1.19	NA	0.08	0.921424
**XCL2**	1.94	1.71	3.55	-0.22	NA	0.81	0.263287

Additional file 4. Expression of chemokine ligands in skin.

HC, healthy control; NA, not available; padj, adjusted p-value; PsA L psoriatic arthritis lesional; PsA U, psoriatic arthritis uninvolved
