# Supplementary material for: Cutaneous lesions in psoriatic arthritis are enriched in chemokine transcriptomic pathways
Source: Arthritis Res Ther. 2023 May 2;25:73. doi: 10.1186/s13075-023-03034-6 (PMC10152590; doi:10.1186/s13075-023-03034-6)
Supplement: Supplementary file 5 — Additional file 5. Expression of chemokine receptors in skin. [file 13075_2023_3034_MOESM5_ESM.pdf]

	Mean normalised read count			PsA U vs HC		PsA L vs HC	
**symbol**	**HC**	**PsA U**	**PsA L**	**log2fold**	**p.adj**	**log2fold**	**p.adj**
**CCR1**	46.64	37.76	138.22	-0.31	0.7479	1.56	0.000494
**CCR2**	65.59	63.24	192.48	-0.05	0.9493	1.55	2.94E-07
**CCR3**	14.65	9.54	6.53	-0.62	NA	-1.17	0.018607
**CCR4**	25.89	38.44	158.00	0.56	0.5620	2.6	8.17E-08
**CCR5**	44.32	37.38	199.95	-0.25	0.7347	2.17	4.41E-11
**CCR6**	3.21	2.15	3.42	-0.6	NA	0.04	0.966631
**CCR7**	29.52	30.61	210.81	0.05	0.9610	2.83	1.09E-19
**CCR8**	8.96	7.55	18.74	-0.25	NA	1.05	0.030999
**CCR9**	1.12	0.81	1.77	-0.59	NA	0.6	0.555578
**CCR10**	16.90	14.49	9.25	-0.24	NA	-0.88	0.011446
**CXCR1**	16.97	43.18	51.12	1.35	0.1201	1.6	0.001303
**CXCR2**	124.43	143.05	871.71	0.2	0.7880	2.81	7.71E-19
**CXCR3**	16.01	14.19	52.47	-0.18	0.8482	1.71	6.1E-06
**CXCR4**	88.15	98.40	584.47	0.15	0.8165	2.72	8.62E-24
**CXCR5**	1.70	0.76	1.13	-1.17	NA	-0.63	0.540701
**CXCR6**	12.65	18.02	174.11	0.52	0.4846	3.8	1.32E-26
**CX3CR1**	94.13	85.80	138.34	-0.13	0.8494	0.56	0.087562
**XCR1**	170.31	198.89	196.92	0.22	0.5627	0.21	0.366734
**ACKR1**	1228.70	1023.04	777.53	-0.26	0.4686	-0.66	0.000765
**ACKR2**	43.73	38.11	455.69	-0.2	0.7324	3.38	9.51E-41
**ACKR3**	834.20	930.09	670.58	0.16	0.8376	-0.32	0.413895
**ACKR4**	237.26	209.36	151.10	-0.18	0.8136	-0.65	0.072549

Additional file 5. Expression of chemokine receptors in skin.

HC, healthy control; NA, not available; padj, adjusted p-value; PsA L psoriatic arthritis lesional; PsA U, psoriatic arthritis uninvolved
