# Supplementary material for: Cutaneous lesions in psoriatic arthritis are enriched in chemokine transcriptomic pathways
Source: Arthritis Res Ther. 2023 May 2;25:73. doi: 10.1186/s13075-023-03034-6 (PMC10152590; doi:10.1186/s13075-023-03034-6)
Supplement: Supplementary file 6 — Additional file 6. The expression of CXCR6 in skin lesions correlates with PASI score. The normalised read counts of CXCR6 are plotted on the y-axis and PASI scores on the x-axis. Spearman r 0.7748, p 0.04921. [file 13075_2023_3034_MOESM6_ESM.pptx]

## Slide 1
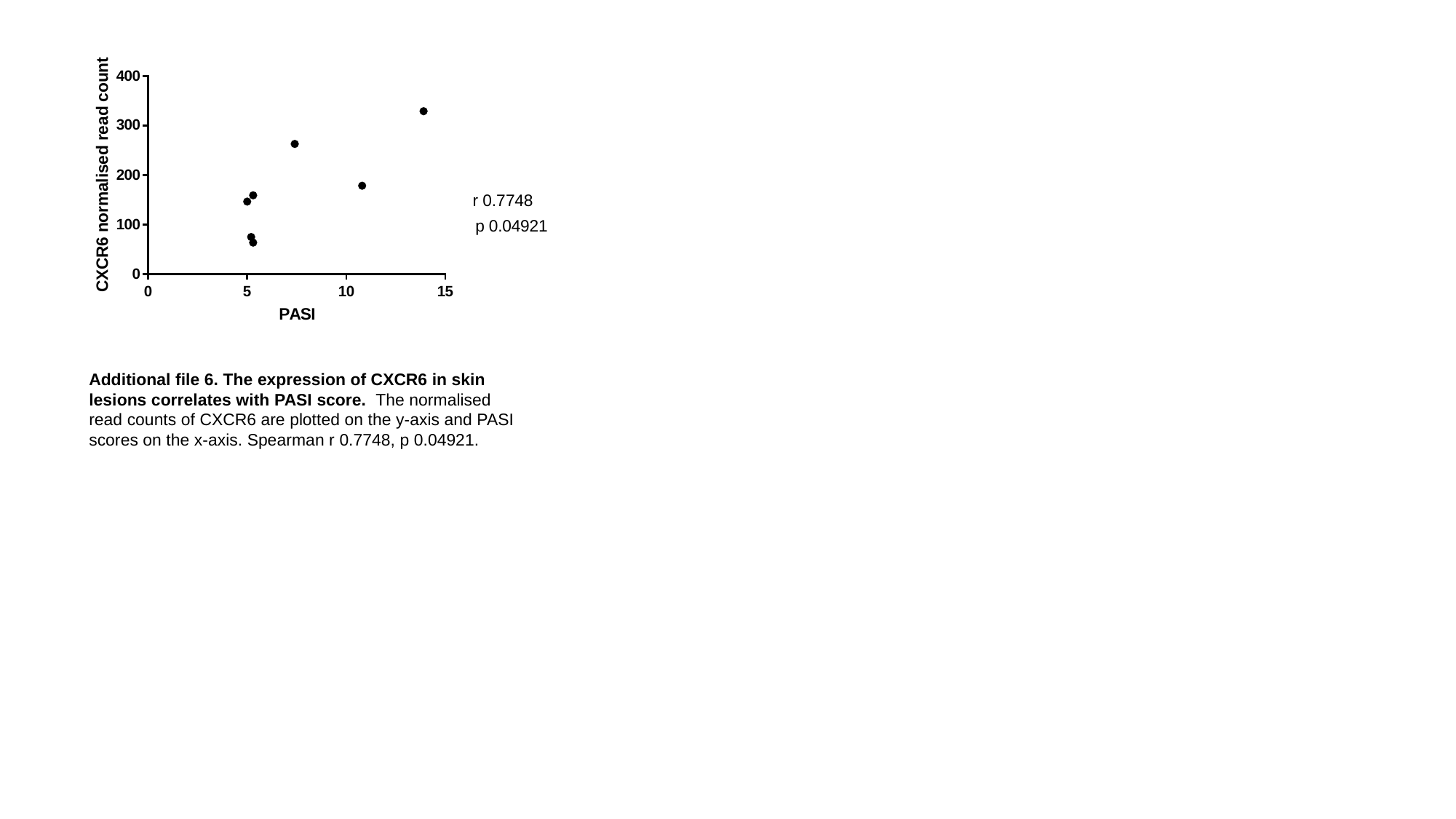

Additional file 6. The expression of CXCR6 in skin lesions correlates with PASI score. The normalised read counts of CXCR6 are plotted on the y-axis and PASI scores on the x-axis. Spearman r 0.7748, p 0.04921.
